# Random-access imaging of awake behaving animals

**DOI:** 10.1038/lsa.2016.275

**Published:** 2017-02-24

**Authors:** Meng Cui

**Affiliations:** 1 School of Electrical and Computer Engineering and Department of Biology, Purdue University, West Lafayette, IN 47907, USA

A compact random-access microscopy system offers inertia-free rapid focusing and line scanning, which enables near simultaneous 3D imaging of neural network in awake behaving animals at high-spatiotemporal resolution.

To understand the function and principles of operation of neural networks, we need to observe the flow of information in the brain at high-spatiotemporal resolution. Over the past two decades, laser scanning two-photon fluorescence microscopy (TPM) combined with sensitive calcium indicators have emerged as the powerhouse for *in vivo* recording of neuronal activity^1^. TPM differs from electrophysiology, in that allows noninvasive measurement of activity at excellent spatial resolution, sufficient for resolving fine structures such as dendritic spines. Moreover, the high throughput of TPM enables the measurement of a large population of neurons within a single recording.

To further improve the throughput, a variety of TPM imaging systems have been developed^2, 3, 4, 5^. As neurons and/or their processes are often sparsely distributed in 3D, the most efficient imaging strategy is to selectively image them and not the space between them. This selective imaging of regions of interest can be achieved with random-access scanning (RAS). For RAS systems, acousto-optic deflector (AOD) is typically employed for its rapid inertia-free 3D scanning. Experimentally, the users first acquire a 3D continuous volumetric image, then define the points of interest and command the AOD to only visit these points in 3D repeatedly over time to record the calcium transients.

However, the brain motion that occurs when imaging awake behaving animals can easily cause measurement artifact. The translation of the tissue with respect to the predefined measurement locations can cause signal variation, which can be misinterpreted as calcium transients. For this reason, RAS has not been widely used for behaving animal studies. In a recent publication, Nadella *et al.*^6^ developed a novel AOD-based RAS system that extends the functionality of this technology and addresses the issue of motion artifacts.

Compared with previous reports, the new RAS system presents several advances. First, the new design employed thin AODs in a compact configuration (Figure 1). The entire assembly of the four AODs that make up a spherical acousto-optic lens was contained in a 20 × 20 × 30 cm^3^ box, which can be inserted as an add-on module to existing TPM systems. Second, the usage of elliptically polarized light and the field-programmable gate array-based AOD control system provides better signal uniformity across a large scanning angle. Third, the synchronized high-speed data acquisition allows rapid line scanning in 3D, especially in the planes that are far away from the natural focal plane of the objective lens. These features provide great flexibility and convenience to the calcium imaging measurement. One can carry out (1) high-speed continuous volumetric imaging, (2) full-frame multi-plane imaging, (3) random-access sub-volume imaging, (4) random-access patch imaging and (5) random-access point imaging.

The random-access sub-volume imaging and patch imaging can nicely handle the motion issue during awake behaving animal imaging. With 2D images or 3D volumes recorded around the features of interest, one can conveniently remove the motion artifact via post-measurement image registration. These capabilities are very powerful for imaging sparsely distributed neurons or monitoring dendritic and somatic activity simultaneously across large depth ranges, which are important for answering many fundamental neuroscience questions.

## Figures and Tables

**Figure 1:**
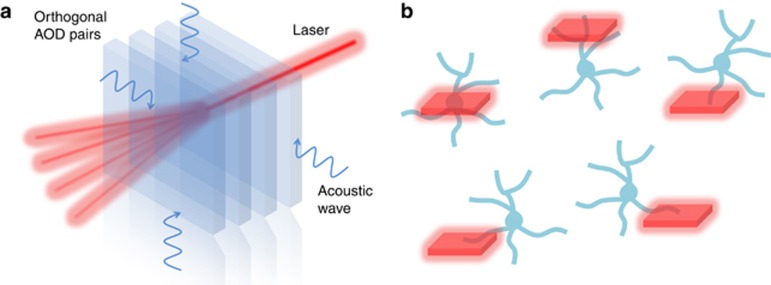
3D random-access imaging of neurons with a compact AOD system. (**a**) The two orthogonally oriented AOD pairs can freely focus the femtosecond laser beam in 3D. (**b**) The synchronized high-speed data acquisition controlled by field-programmable gate array allows rapid random-access sub-volume imaging and patch imaging of neurons in awake behaving animals.

## References

[bib1] Svoboda K, Yasuda R. Principles of two-photon excitation microscopy and its applications to neuroscience. Neuron 2006; 50: 823–839. 16772166 10.1016/j.neuron.2006.05.019

[bib2] Duemani Reddy G, Kelleher K, Fink R, Saggau P. Three-dimensional random access multiphoton microscopy for functional imaging of neuronal activity. Nat Neurosci 2008; 11: 713–720. 18432198 10.1038/nn.2116 PMC2747788

[bib3] Grewe BF, Langer D, Kasper H, Kampa BM, Helmchen F. High-speed *in vivo* calcium imaging reveals neuronal network activity with near-millisecond precision. Nat Methods 2010; 7: 399–405. 20400966 10.1038/nmeth.1453

[bib4] Katona G, Szalay G, Maák P, Kaszás A, Veress M et al. Fast two-photon *in vivo* imaging with three-dimensional random-access scanning in large tissue volumes. Nat Methods 2012; 9: 201–208. 22231641 10.1038/nmeth.1851

[bib5] Kong L, Tang J, Little JP, Yu Y, Lämmermann T et al. Continuous volumetric imaging via an optical phase-locked ultrasound lens. Nat Methods 2015; 12: 759–762. 26167641 10.1038/nmeth.3476 PMC4551496

[bib6] Nadella KNS, Roš H, Baragli C, Griffiths VA, Konstantinou G et al. Random-access scanning microscopy for 3D imaging in awake behaving animals. Nat Methods 2016; 13: 1001–1004. 27749836 10.1038/nmeth.4033 PMC5769813

